# Bioinformatics meets machine learning: identifying circulating biomarkers for vitiligo across blood and tissues

**DOI:** 10.3389/fimmu.2025.1543355

**Published:** 2025-05-15

**Authors:** Qiyu Wang, Jingwei Yuan, Mengdi Zhang, Haiyan Jia, Hongjie Lu, Yan Wu

**Affiliations:** ^1^ Beijing Technology and Business University, Beijing, China; ^2^ Air Force Medical Center of the Chinese People’s Liberation Army, Beijing, China

**Keywords:** vitiligo, pigmentation loss, WGCNA, machine learning, immune infiltration, biomarkers

## Abstract

**Background:**

Vitiligo is a skin disorder characterized by the progressive loss of pigmentation in the skin and mucous membranes. The exact aetiology and pathogenesis of vitiligo remain incompletely understood.

**Methods:**

First, a microarray dataset of blood samples from multiple patients with vitiligo was collected from GEO database.The limma package was used to analyze the microarray data and identify significant differentially expressed genes (DEGs). The merged microarray data were then used for WGCNA to identify modules of features genes. DEGs selected with the limma package and module genes derived from the WGCNA were intersected using the Venn package in R. Enrichment analyses were performed on the overlapping genes, including Gene Ontology and Kyoto Encyclopedia of Genes and Genomes methodology. Advanced screening was performed using the least absolute shrinkage and selection operator and support vector machine techniques from the machine learning toolkit. CIBERSORT was used to analyse the immune cell composition in the microarray data to assess the relationships among these genes and immune cells. Biological samples were obtained from the patients, and gene expression analysis was performed to evaluate the levels of core genes throughout the progression of vitiligo. Finally, we obtained the microarray datasets GSE53146 and GSE75819 from the affected skin of vitiligo patients and GSE205155 from healthy skin to perform expression analysis and gene set enrichment analysis of the hub genes.

**Results:**

Two hub genes, *HMGA1* and *PSMD13*, were identified via machine learning and WGCNA. The analysis of immune cell infiltration suggested that different immune cell types could play a role in the progression of vitiligo. Moreover, these hub genes exhibited varying degrees of association with immune cell profiles. qRT–PCR analysis of blood samples from vitiligo patients revealed notable downregulation of the hub genes. Analysis of the microarray datasets derived from skin lesions revealed that *HMGA1* expression levels remained relatively stable, whereas *PSMD13* expression levels markedly decreased.

**Conclusion:**

*PSMD13* may influence vitiligo development via the Nod-like receptor signaling pathway and could serve as a potential diagnostic marker for evaluating skin lesions in vitiligo.

## 1 Introduction

Vitiligo is an autoimmune dermatological disorder characterized by the loss of melanocytes, the cells that produce skin pigments, leading to areas of depigmentation that manifest as white patches on the skin (1, 2). Statistics indicate that the global incidence rate is 0.36%. In adults, the rate is around 0.67%, while in children it is about 0.24%. Overall, approximately 28.5 million people are affected (3). In addition, due to its negative impact on patients’ social interactions, vitiligo leads to a high prevalence of mental disorders such as anxiety and depression (4, 5), which leads to a significant decline in the quality of life of patients.

The pathogenesis of this disease has not been fully elucidated, and current research has focused primarily on oxidative stress promoting the CD8+ T-cell response in the skin (6, 7). Inherent lymphocytes mediate the apoptosis of melanocytes (8) and the effects of family genetics (9). Present treatments include ultraviolet light–based combination therapies (10), targeted T-cell therapies (11) and JAK inhibitors, but the likelihood of achieving long-term colour recovery is quite small (12). Therefore, investigating the mechanisms underlying vitiligo, identifying biomarkers for this disease, and developing prevention and treatment strategies on the basis of these findings are highly important.

In recent years, advancements in bioinformatics have led to its widespread application along with machine learning techniques for identifying treatment targets for various diseases (13).Gene chips, a high-sequence technique for analysing RNA expression, have been used in medical research into numerous disorders, including neurodevelopmental disorders (NDDs) (14).

Weighted gene co-expression network analysis (WGCNA) is a powerful method for analysing transcriptomic data. In WGCNA, tightly connected genes are clustered into different modules, not only identifying differentially expressed genes (DEGs) but also exploring the correlations between modules and diseases (15). The identification of disease biomarkers through scale-free WGCNA has been utilized in numerous disease studies (16–18). Machine learning encompasses a range of mathematical techniques designed to extract insights from large datasets (19). Machine learning algorithms offer several advantages, such as non-linearity, fault tolerance, and real-time operation, that make them well suited for complex applications (20).

**Figure 1:**
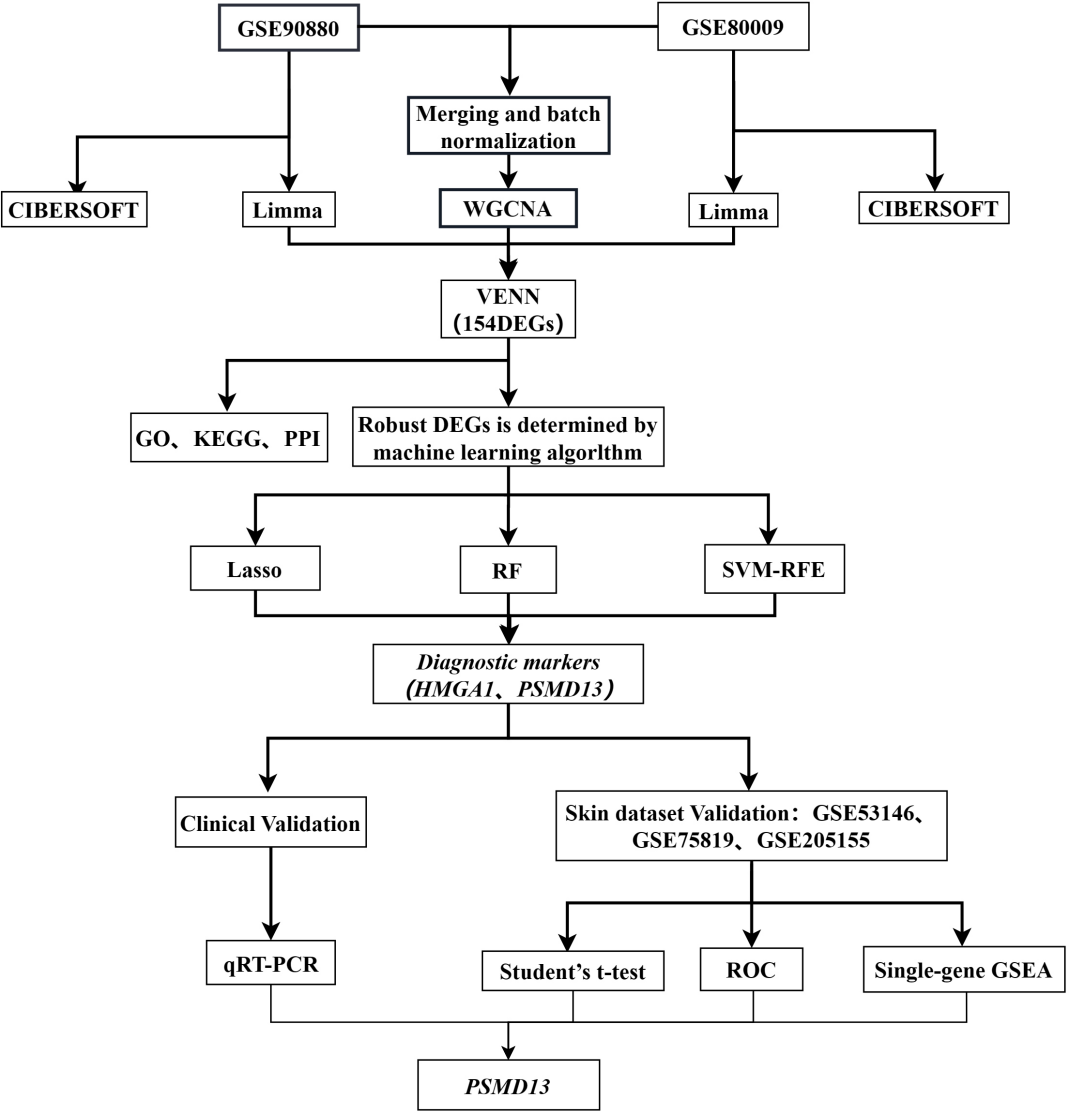
Study flowchart.

In this study, we obtained two expression microarray datasets of peripheral blood mononuclear cells (PBMCs) from vitiligo patients and healthy controls from the Gene Expression Omnibus (GEO) database. Differential gene identification (n=154) was conducted with the limma and WGCNA packages in R. Machine learning algorithms were used to select two core genes. Additionally, we assessed the proportions of 22 immune cell types in the PBMCs of vitiligo patients and healthy individuals via the CIBERSORT algorithm. Biological samples were collected, and the expression levels of these core genes throughout vitiligo pathogenesis were evaluated. Finally, we collected microarray datasets from lesional skin and healthy skin for expression analysis and gene set enrichment analysis (GSEA) of the hub genes. The analysis process of this study is illustrated in **Figure 1**.

## 2 Materials and methods

### 2.1 Gene expression profile (dataset download and processing)

Data were collected from the GEO database (https://www.ncbi.nlm.nih.gov/geo/), a public resource for high-throughput gene expression data, chips, and microarrays. We obtained two datasets derived from blood samples from vitiligo patients: GSE90880 (GPL8300), which included nine vitiligo patients and six healthy controls, and GSE80009 (GPL16951), which included eight vitiligo patients and four healthy controls. Three datasets derived from skin samples of vitiligo patients and healthy controls were used for data validation: GSE53146 (GPL14951), which included 5 vitiligo patients and 5 healthy controls; GSE75819 (GPL6884), which included 15 vitiligo patients; and GSE205155 (GPL14550), which included 11 healthy controls.

### 2.2 Identification of DEGs

We compared vitiligo subjects with healthy control subjects using R (v4.2.2). The R package “limma” was used to identify DEGs between the vitiligo patient group and healthy control group for both the GSE90880 and GSE80009 datasets. Adjusted p values were evaluated to mitigate the risk of false positives in the GEO dataset. DEGs with adjusted p values < 0.05 and |log2 fold change (log2FC)| > 0.5 were deemed significant. Volcano maps were created with a volcano plotting tool (http://soft.sangerbox.com/). Heatmaps based on filtered DEGs were generated with the pheatmap package in R.

### 2.3 Weighted gene co-expression networks

The gene expression profile was generated using the “sva” package to normalize the data and correct for batch effects. The GSE90880 and GSE80009 datasets were subsequently merged. The Pearson correlation coefficient was computed with the R WGCNA software package to determine the correlation between gene pairs, establishing the gene co-expression matrix. Following the scale-free network principle, soft thresholds (power = 12, R^2^ = 0.89) were chosen successively to construct scale-free co-representation networks. The adjacency matrix was then converted into a topological overlap matrix. Subsequently, cluster analysis was performed to delineate gene modules, each containing at least 60 genes. Hierarchical clustering was employed to generate a dendrogram, evaluating the correlation between module feature genes and the disease phenotype. The module with the highest correlation coefficient and the lowest P value was identified as the disease feature. Next, the module feature genes obtained using limma were intersected with the GSE90880 and GSE80009 datasets, and a Venn diagram was generated to identify the hub genes.

### 2.4 Selection of feature genes for Gene Ontology and Kyoto encyclopedia of genes and genomes functional enrichment analyses

The intersecting genes identified by limma and WGCNA were selected using the Venn software package in R.

In this study, the “clusterProfiler” R package was used to perform GO and KEGG functional enrichment analyses, with a focus on gene-related biological process (BP), molecular function (MF), and cellular component (CC) terms and their associated signalling pathways.

### 2.5 Machine learning analysis of disease genes

We employed three machine learning algorithms, namely, least absolute shrinkage and selection operator (LASSO) regression analysis, support vector machine with recursive feature elimination (SVM-RFE) analysis, and random forest (RF) analysis, to analyse the genes identified by the aforementioned methods. We used performed the LASSO regression analysis with the R package “rms”, SVM-RFE analysis using the R package “e1071”, and RF analysis with the R package “randomForest.” The intersecting genes obtained from these three analyses are considered the core genes of vitiligo. Finally, we implemented stratified 5-fold cross-validation in R software to evaluate the logistic regression model built on core genes. Each fold preserved the original class distribution of the dataset. After training the model on four folds, we generated predictions for the held-out test fold and merged all test fold predictions to compute the pooled AUC. To evaluate stability, we conducted 1000 bootstrap resampling on the combined predictions, reporting the mean AUC and the 95% confidence interval.

### 2.6 Immune infiltration analysis

CIBERSORT is a computational tool that was used to estimate the proportions of immune cells in vitiligo and control samples on the basis of tissue gene expression profiles, helping to identify variations in immune cell composition. We analysed immune cell infiltration with the “Cibersort” R software package. Bar graphs illustrating the distributions of various immune cell types across different samples were generated. The proportions of different immune cell types in the vitiligo and control groups were compared with violin plots. Additionally, heatmaps showing the correlations among 22 types of infiltrating immune cells were generated with the “corrplot” R package.

### 2.7 Clinical validation of core genes

#### 2.7.1 Sample collection

This study received approval from the Medical Ethics Committee of the Air Force Medical Center of the Chinese People’s Liberation Army (Approval No. 2023-67-PJ01) and followed ethical guidelines consistent with the principles established by the Declaration of Helsinki. All participants provided written informed consent. A total of 30 patients with vitiligo and 30 healthy controls participated in this study. Our research primarily focuses on individuals with the disease who have not undergone systemic treatment. The inclusion criteria are based on the consensus for diagnosing and treating vitiligo (21).: (1) skin lesions presented as depigmented white patches of varying sizes and shapes, with normal or increased pigmentation around the edges; (2) skin lesions commonly occurred on the face, neck, hands, torso, and oral and mucosal tissues and the surrounding skin and were frequently found in traumatized areas, with hairs in the white patches typically turning white; (3) other pigmentation disorders or depigmentation diseases were excluded; (4) bright white fluorescence was observed in the white patches under Wood’s lamp examination; (5) disease duration of over 2 years; (6) absence of comorbid autoimmune diseases; and (7) no history of systemic or immunosuppressive therapies.

#### 2.7.2 RNA extraction and quantitative RT–PCR

Total RNA was extracted from peripheral blood using a whole-blood total RNA extraction kit (Simgen, China). The concentration and purity of the extracted RNA were assessed with a NanoDrop 2000 spectrophotometer (Thermo Fisher Scientific, Waltham, MA, USA). The assessment focused on A260/A280 ratios that fell within the range of 1.8 to 2.0. The primer sequences of the hub genes used in qRT-PCR are shown in **Table 1**.

**Table 1:** Sequences of the primers utilized for quantitative real-time PCR.

Gene	Forwards primer sequence	Reverse primer sequence
*HMGA1*	AGCGAAGTGCCAACACCTAAG	TGGTGGTTTTCCGGGTCTTG
*PSMD13*	CAGATGACTGATCCTAATGTGGC	CCAGGAAGGTTGTTGAGCATTT
*GAPDH*	GGCACAGTCAAGGCTGAGAATG	ATGGTGGTGAAGACGCCAGTA

### 2.8 Skin dataset validation of the hub genes

#### 2.8.1 Merging multiple datasets and removing batch effects

We first merged GSE53146 (GPL14951), GSE75819 (GPL6884) and GSE205155 (GPL14550) using the inSilicoMerging package in R(A total of 18 cases of vitiligo patients and 12 healthy controls) (22), followed by the Combat package to remove batch effects (23).

#### 2.8.2 Expression of the hub genes in the skin-derived datasets

The expression of the hub genes in the skin-derived datasets was calculated via t tests.

#### 2.8.3 Receiver operating characteristic curve analysis

The diagnostic performance of the hub genes was assessed via ROC curve analysis.

#### 2.8.4 Single-gene GSEA

We conducted single-gene GSEA to explore potential pathways regulated by the hub genes.

### 2.9 Statistical analysis

GraphPad Prism (version 9.5.0, San Diego, California, USA) was used to perform two-sided Student’s t tests to compare the differences between the disease group and the normal group. The t value and P value for each gene were calculated. A P value less than 0.05 was considered to indicate a significant difference between the normal group and the vitiligo group.

## 3 Results

### 3.1 Identification of DEGs

**Figure 2:**
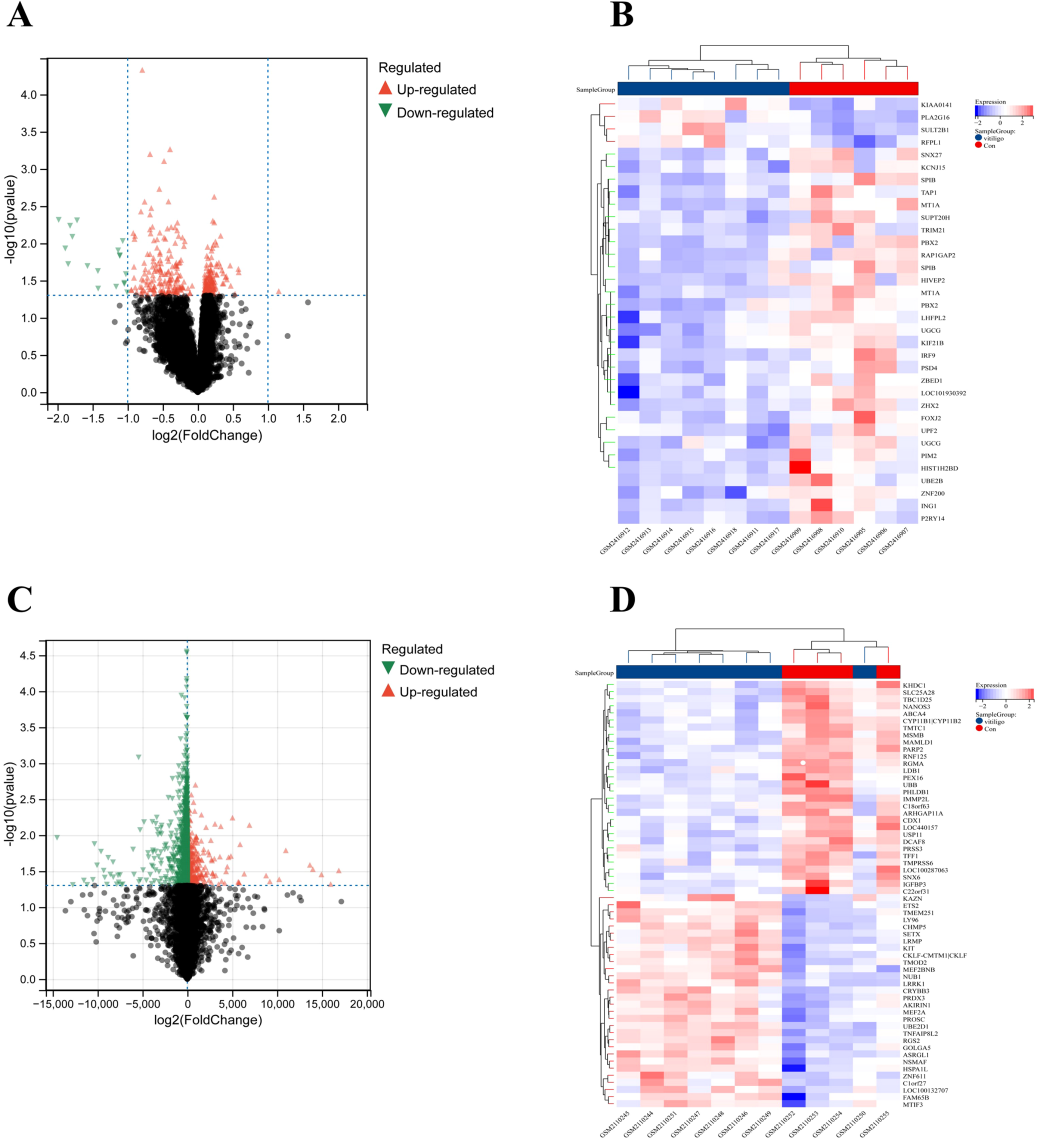
Dysregulated genes in vitiligo are displayed in **(A)** the volcano plot and **(B)** the heatmap for GSE90880 and in **(C)** the volcano plot and **(D)** the heatmap for GSE80009.

In this study, we identified 347 DEGs, including 154 that were upregulated and 193 that were downregulated, from a dataset consisting of 8,621 samples. This dataset included 4 blood samples from healthy individuals and 8 blood samples from patients with vitiligo, sourced from GSE90880. Additionally, 2423 DEGs (398 upregulated genes and 2025 downregulated genes) were selected from a dataset of 20134 samples (including 6 blood samples from healthy individuals and 8 blood samples from vitiligo patients) obtained from GSE80009. A volcano plot of the DEGs was created with thresholds of log2FC greater than 0.5 and an adjusted p value less than 0.05 (**Figures 2A, C**). Additionally, a heatmap was generated to illustrate the top 30 upregulated and top 30 downregulated genes (**Figures 2B, D**). 

### 3.2 Weighted gene co-expression network analysis

**Figure 3:**
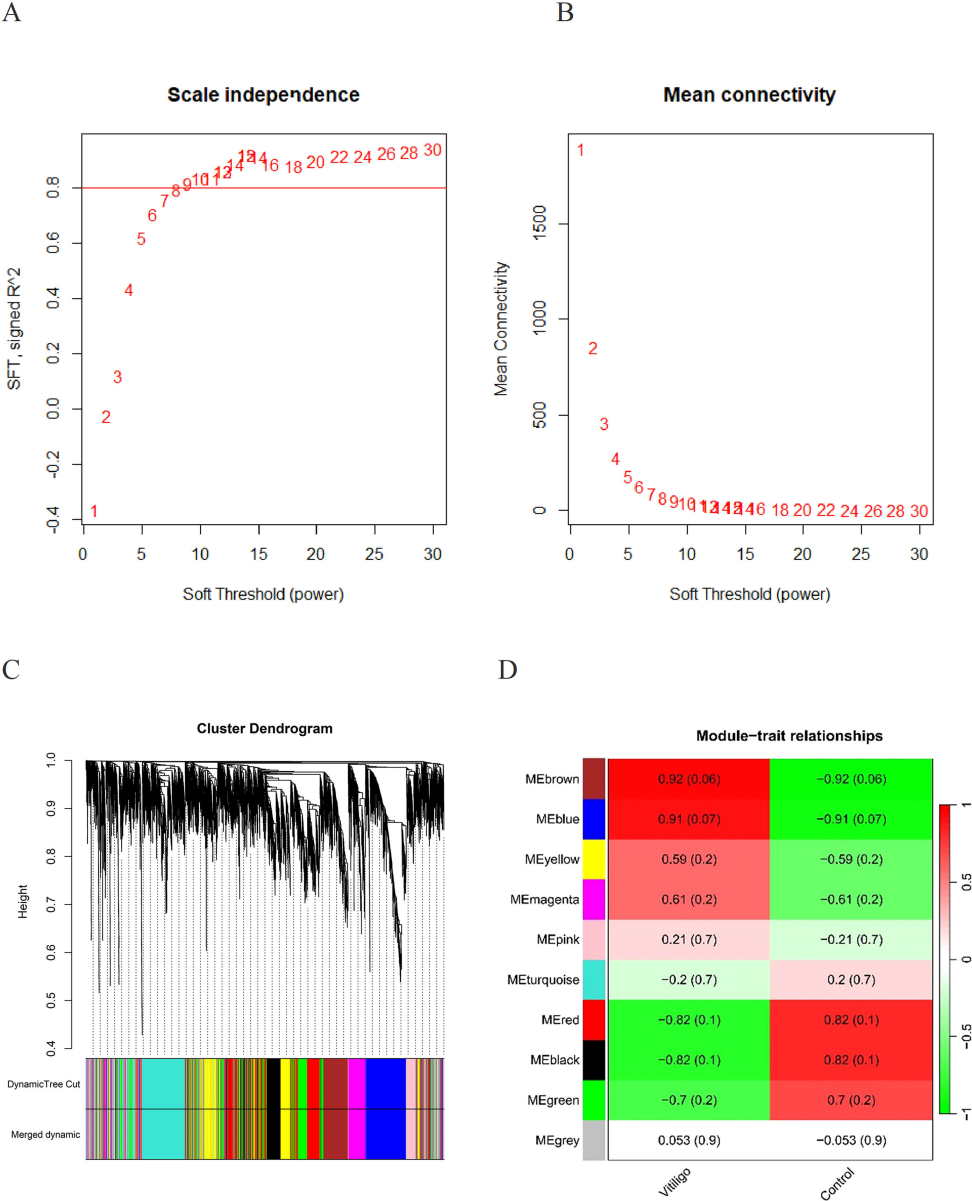
**(A)** Determination of the appropriate soft threshold for constructing a scale-free gene co-expression network. **(B)** Hierarchical clustering representation of all genes. **(C)** Cluster dendrograms showed the clustering process of the gene modules. **(D)** Assessment of the relationship between individual modules and vitiligo patients versus controls.

We utilized WGCNA to detect gene modules associated with specific traits. Initially, we calculated a matrix of similarities and transformed it into an adjacency matrix, applying an optimal soft threshold (β = 12) (**Figures 3A, B**). Ten distinct gene modules were identified in the analysis (**Figure 3C**). We identified the “brown” module (correlation coefficient = 0.92, *p*-value = 0.06) and the “blue” module (correlation coefficient = 0.91, *p*-value = 0.07) as the modules most clinically relevant to vitiligo on the basis of their associations with the phenotypic traits of the condition (**Figure 3D**).

The intersection of the DEGs identified by limma in GSE90880 and GSE80009 with the key module genes identified by WGCNA via Venn analysis resulted in the identification of 154 core genes.

**Figure 4:**
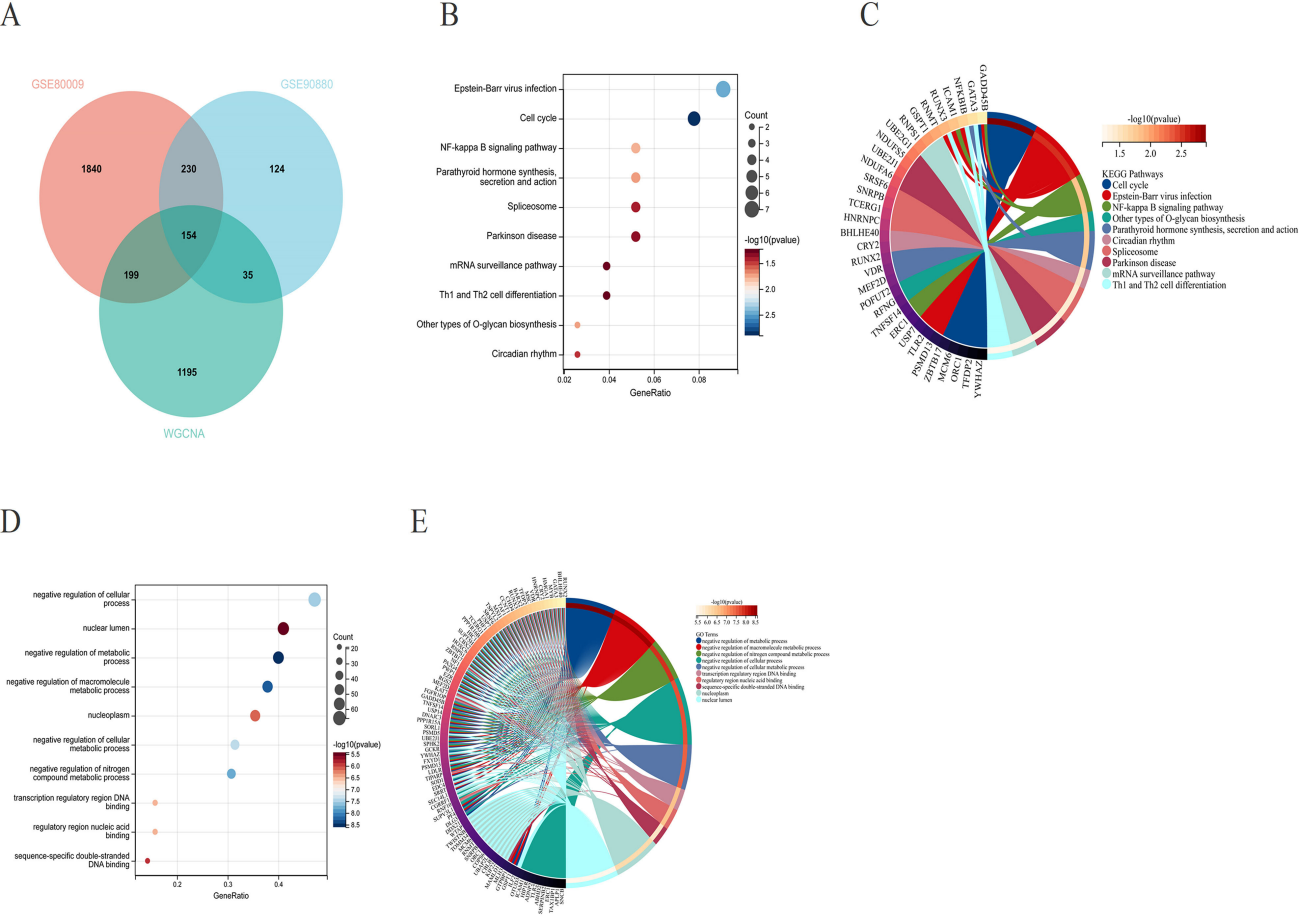
**(A)** Venn diagram illustrating the overlap between the differentially expressed genes from GSE90880 and GSE80009 and the key module genes identified through WGCNA, revealing a total of 154 core genes. **(B, C)** Kyoto Encyclopedia of Genes and Genomes (KEGG) pathway analysis of the core genes. **(D, E)** Gene Ontology (GO) analysis of the core genes to assess their functional characteristics.

To examine the regulatory roles of the 154 core genes in disease development, we compared the DEGs from GSE90880 with those from GSE80009, which were identified via the limma package, along with key module genes identified via WGCNA, ultimately leading to the selection of the 154 core genes (**Figure 4A**). We subsequently conducted GO and KEGG pathway analyses on the 154 core genes to identify the biological functions of these DEGs. KEGG analysis revealed that the DEGs were associated primarily with pathways such as Epstein–Barr virus infection; the cell cycle; the NF-κB signalling pathway; parathyroid hormone synthesis, secretion and action; and the spliceosome (**Figures 4B, C**). GO analysis revealed enrichment of DEGs in pathways related to negative regulation of cellular processes, the nuclear lumen, negative regulation of metabolic processes, negative regulation of macromolecule metabolic processes, the nucleoplasm, and negative regulation of cellular processes (**Figures 4D, E**).

### 3.3 Machine learning

**Figure 5:**
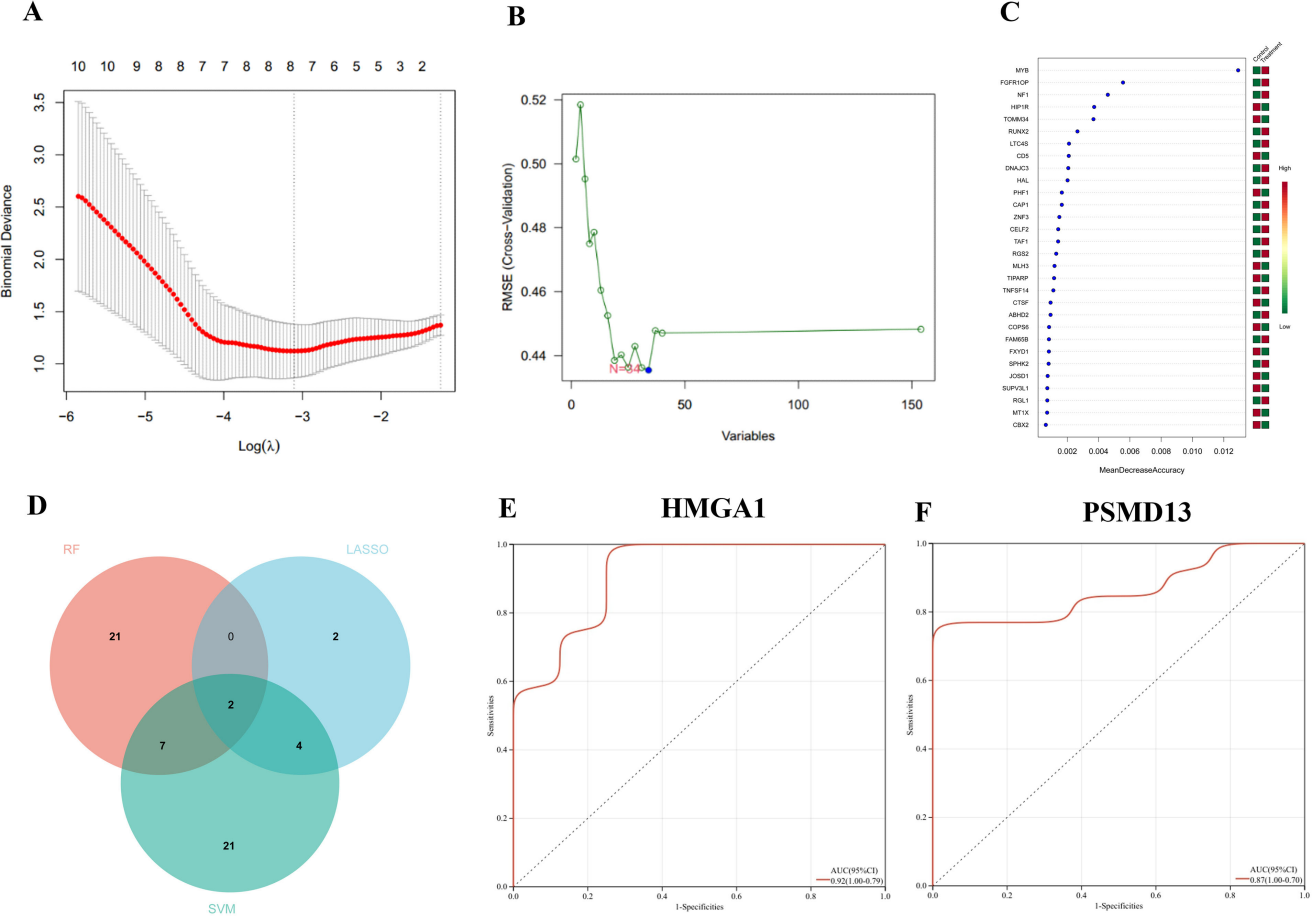
Machine learning algorithms: **(A)** least absolute shrinkage and selection operator (LASSO) regression, **(B)** support vector machine with recursive feature elimination (SVM-RFE), and **(C)** the random forest (RF) algorithm. **(D)** Venn diagram illustrating the overlapping and distinct genes identified by LASSO, SVM-RFE, and RF. **(E, F)** ROC curve of hub genes *HMGA1* and *PSMD13*.

First, the LASSO regression algorithm was used to identify 16 hub genes (**Figure 5A**). Next, the SVM-RFE algorithm was subsequently employed to identify 8 hub genes (**Figure 5B**). The RF algorithm was used for identification (**Figure 5C**). We visualized the overlapping hub genes from the three algorithms using a Venn diagram, which revealed 2 hub genes: HMGA1 and PSMD13 (**Figure 5D**). Finally, we conducted ROC curve analysis on the 2 hub genes, yielding an AUC of 0.92 for *HMGA1* and 0.87 for *PSMD13* (**Figures 5E, F**).

### 3.4 CIBERSORT

**Figure 6:**
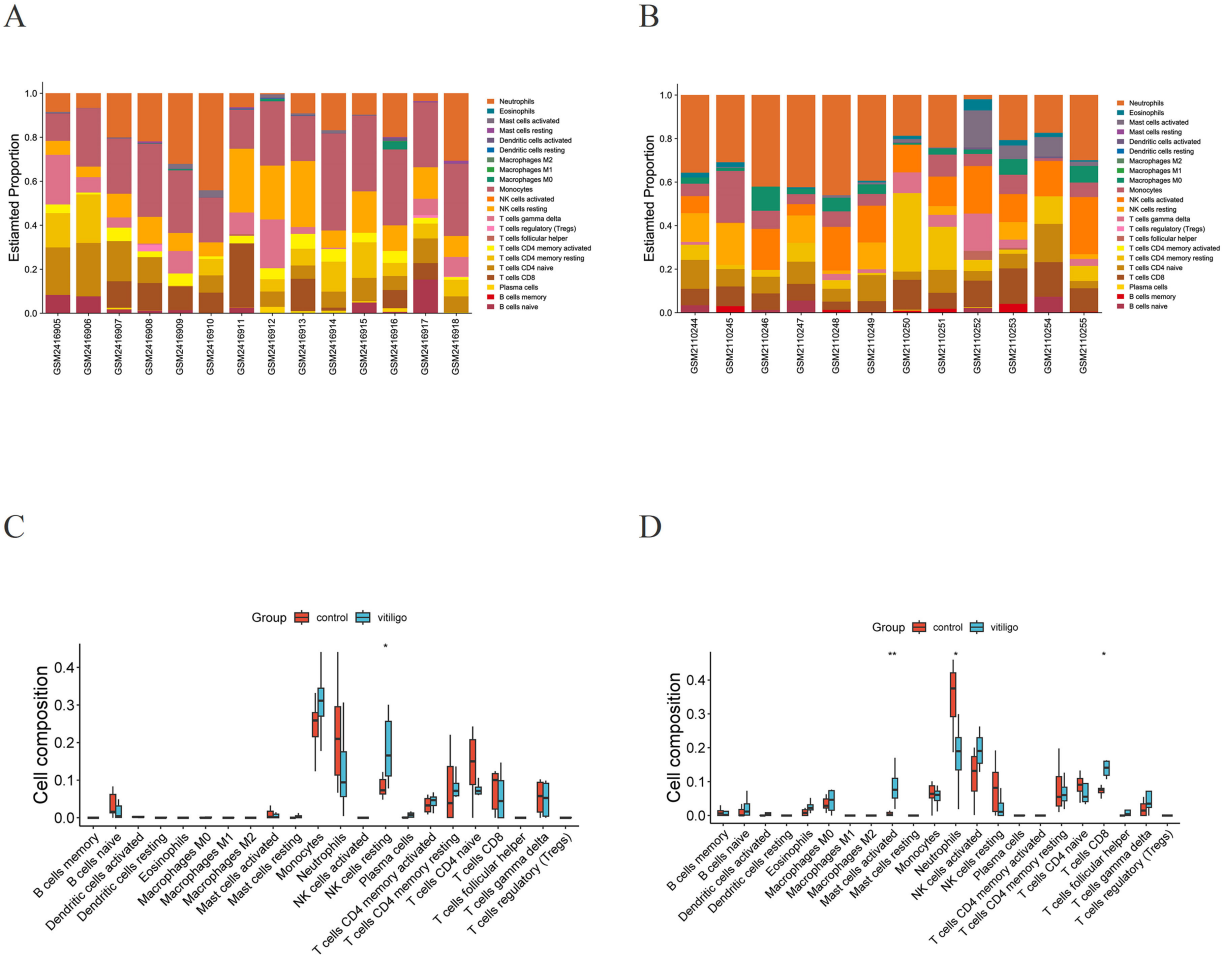
Immune infiltration analysis. **(A)** Stacked bar plot visualization of various infiltrating immune cells in GSE90880. **(B)** Stacked bar plot visualization of various infiltrating immune cells in GSE80009. **(C)** Violin plot visualization of infiltrating immune cells in the vitiligo and normal groups in GSE90880. *p < 0.05, **p < 0.01. Wilcoxon rank-sum test. **(D)** Violin plot visualization of infiltrating immune cells in the vitiligo and normal groups in GSE80009. *p < 0.05, **p < 0.01. Wilcoxon rank-sum test.

Because the hub genes were found to be enriched in immune-related pathways, we explored the immune landscape in the training datasets via the CIBERSORT algorithm to better elucidate the role of immune regulation in vitiligo pathogenesis. **Figure 6** shows the infiltration levels of 22 immune cell types in both the vitiligo and normal groups. The bar plot visually depicts the proportions of immune cells in each sample from the GSE90880 and GSE80009 datasets (**Figures 6A, B**). The violin plots displaying differences in immune cell infiltration revealed that, compared with normal control samples, resting natural killer (NK) cells (p <0.05) exhibited greater infiltration in the GSE90880 dataset (**Figure 6C**). In the GSE80009 dataset, mast cells were activated (p <0.01), and neutrophils (p <0.05) had increased infiltration (**Figure 6D**).

### 3.5 qRT–PCR

**Figure 7:**
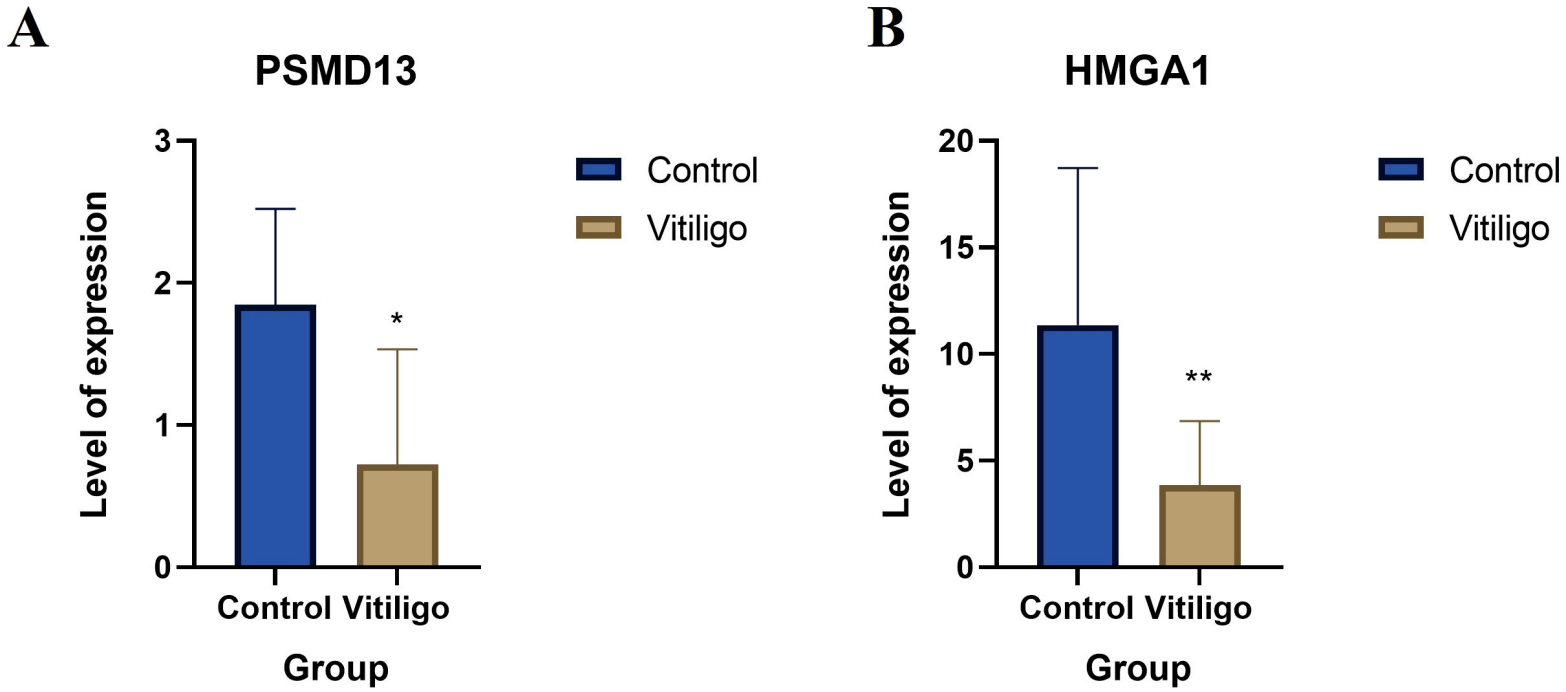
Clinical validation of the hub genes. **(A)** Relative mRNA levels of *HMGA1* in controls and vitiligo patients. **(B)** Relative mRNA levels of *PSMD13* in controls and vitiligo patients (* *p* < 0.05, ** *p* < 0.01).

The expression levels of the overlapping hub genes *HMGA1* and *PSMD13* in the peripheral blood of vitiligo patients and control patients were assessed via qRT–PCR. *HMGA1* and *PSMD13* expression levels were significantly lower in the vitiligo group than in the control group (**Figure 7A**, *P*=0.015; **Figure 7B**, *P*=0.0077), which is consistent with the results of the bioinformatics analysis.

### 3.6 Validation of the hub genes in the skin-derived dataset

**Figure 8:**
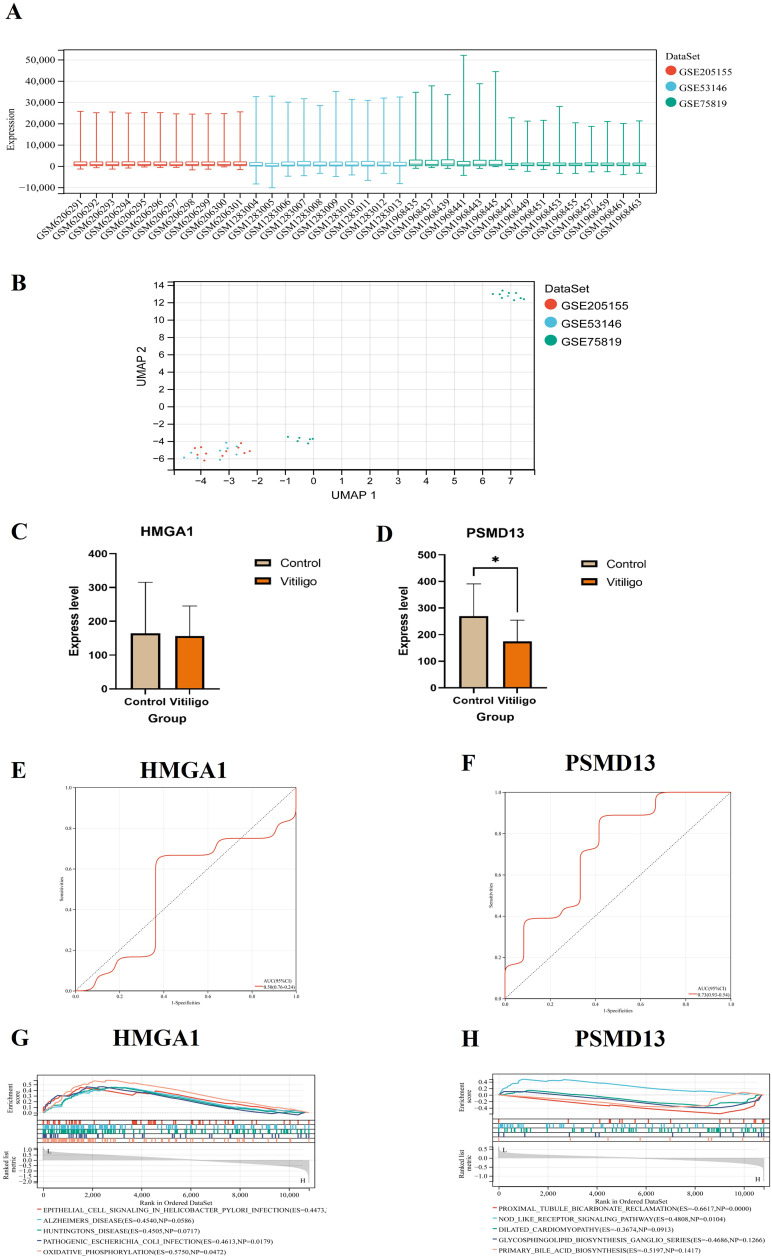
Validation of the hub genes in the skin-derived dataset. **(A)** Boxplot after removal of the batch effect. **(B)** UMAP plot after removal of the batch effect. **(C, D)** Expression of hub genes and validation of skin-derived datasets (* *p* < 0.05). **(E, F)** Hub genes in the skin-derived datasets were analysed via ROC curves. **(G, H)** GSEA of the hub genes.

After the batch effect was removed, a boxplot was generated, which suggested that the data distribution among the datasets converged and that the median was on a line (**Figure 8A**), and the uniform manifold approximation and projection (UMAP) plot demonstrated that the samples from the different datasets clustered closely together, indicating effective removal of the batch effect (**Figure 8B**). The changes in *HMGA1* expression in the inflamed skin of vitiligo patients compared with that in healthy skin were not significant, but *PSMD13* expression showed a significant downwards trend (**Figures 8C, D**). We conducted ROC curve analysis to evaluate the diagnostic potential of the two hub genes, with an area under the curve (AUC) value greater than 0.7 considered to indicate a potential diagnostic marker. In the combined dataset, the AUC value of *HMGA1* was 0.50, and that of *PSMD13* was 0.73 (**Figures 8E, F**). GSEA revealed that the group with low *HMGA1* expression was enriched in epithelial cell signalling pathways associated with *Helicobacter pylori* infection (**Figure 8G**). The group with low *PSMD13* expression was significantly enriched in the NOD-like receptor signalling pathway and highly enriched in the proximal tubule bicarbonate reclamation pathway (**Figure 8H**).

## 4 Discussion

The pathogenesis of vitiligo is closely related to autoimmune and oxidative stress (1, 24). This study identified two key genes, HMGA1 and PSMD13, in the blood of vitiligo patients using bioinformatics and machine learning techniques. These genes were found to be downregulated in vitiligo patients and may trigger the onset of the disease via the NOD-like receptor and NF-κB signaling pathways. Additionally, the expression of these key genes was confirmed using clinical blood samples and skin datasets. These findings indicate that these genes may significantly contribute to the pathogenesis of vitiligo.

Nucleotide-binding and oligomerization domain (NOD)-like receptors (NLRs) are intracellular proteins with a central role in innate and adaptive immunity (25). NLRs include inflammasome receptors/sensors leading to the maturation of caspase 1, IL-1β, IL-18, and gasdermin D to drive inflammation and cell death (26). Additionally, several members of the NLR family act as checkpoints in innate immunity by functioning as negative regulators. Various NLRs regulate the balance between cell death, cell survival, autophagy, mitophagy, and even cellular metabolism (27). Many preclinical studies have confirmed that NLRs are closely related to the pathogenesis of vitiligo, indicating that NLRs can regulate melanocyte apoptosis, thereby mediating the onset of vitiligo (28–31). The NF-κB pathway is crucial for inducing pro-inflammatory cytokines, chemokines, and other inflammatory mediators in different types of innate immune cells. These inflammatory mediators can cause inflammation directly or indirectly promote the differentiation of inflammatory T cells (32). Research indicates that modulating the NF-κB pathway may alleviate melanocyte damage, offering new insights for the clinical management of vitiligo (33, 34). Additionally, NLRs can respond to external signals to activate NF-κB signaling. They negatively regulate NF-κB signal transduction by interacting with TNF receptor-associated factor 6 (TRAF6), which results in the secretion of inflammatory factors (35, 36).

*PSMD13* (26S proteasome non-ATPase regulatory subunit 13), also known as S11 or Rpn9, is a regulatory subunit of the 26S proteasome. *PSMD13* facilitates the ATP-dependent degradation of ubiquitinated proteins, which is crucial for maintaining cellular function through the removal of damaged proteins, and it is also a fundamental complex that helps regulate T-cell function (37). *PSMD13* is involved in processes such as cell cycle regulation and DNA replication, which are essential for maintaining cellular health and longevity (38, 39). One study revealed that *PSMD13* inhibits the NF-κB pathway, indicating its significant role in innate immune responses (40, 41). In addition, *PSMD13* is involved in the ageing process and is related to the human lifespan (42). A decrease in *PSMD13* expression weakens the resistance of cells to emergency injury and megakaryocyte differentiation (43). Our study revealed a decrease in *PSMD13* expression within the blood samples of vitiligo patients, corroborated by a consistent downwards trend observed in follow-up validations using lesional skin datasets. Further insights gained from single-gene GSEA of patient lesional skin datasets implicate the potential role of the Nod-like receptor signalling pathway in the manifestation of characteristic skin lesions in vitiligo patients. TAK1 plays a crucial role in the NF-κB signaling pathway. It can induce pyroptosis by facilitating the nuclear translocation of NF-κB p65 and activating the NOD-like receptor pyrin domain-containing 3 (NLRP3) inflammasome (44, 45). Studies have demonstrated that *PSMD13* inhibits the development of NF-κB signaling pathway neuroinflammation by targeting TAK1 (40). In summary, *PS*MD*13* may inhibit NF-κB signaling through the degradation of TAK1 or kinases associated with the Nod-like receptor pathway. Additionally, its proteasome function might regulate the stability of NLRs, which could reduce melanocyte damage caused by the inflammasome.

The *HMGA1* protein is a nuclear architectural factor belonging to the non-histone chromosomal binding protein superfamily. *HMGA1* can resist cell apoptosis and enhance senescence, thereby preventing replication (46, 47). Its downregulation can promote myocyte differentiation and skeletal muscle regeneration (48), potentially serving as a downstream target of DNA damage (49). On the one hand, the protein encoded by the *HMGA1* gene regulates gene transcription activity and chromatin structure in the nucleus and plays an important role in cell proliferation, differentiation and growth (50, 51). Our results suggest that in vitiligo, the abnormal expression of *HMGA1* may lead to changes in the function of pigment cells, increasing their vulnerability to attack by immune cells or triggering an autoimmune response, thus exacerbating vitiligo. In addition to the NF-κB signalling pathway, the Epstein–Barr virus infection pathway is also relevant to the pathogenesis of vitiligo according to KEGG analysis. Although this pathway is associated primarily with viral infection, its role in immune regulation and cell proliferation processes may indirectly influence the immunopathological mechanisms of vitiligo (52). On the other hand, the *PSMD13* gene encodes a protein involved in regulating the cell cycle and the NF-κB signalling pathway, both of which are critical in immune responses and apoptosis (41). The NF-κB signalling pathway plays a crucial role in regulating inflammation, and its abnormal activation is associated with various autoimmune diseases, including vitiligo. Studies indicate that aberrant activation of the NF-κB signalling pathway may lead immune cells to attack melanocytes, affecting their normal function and promoting the onset and progression of vitiligo (33, 53).

The role of immune cells in vitiligo cannot be overlooked (54). NK cells, activated mast cells, neutrophils, and other immune cells play crucial roles in the process of immune inflammation. These cells can influence the function and survival of melanocytes directly or indirectly through the release of inflammatory mediators and the activation of immune responses, thereby participating in the pathological processes of vitiligo (55–57).

Our analysis of blood microarray data revealed that reduced expression levels of the *PSMD13* and *HMGA1* genes influence cellular processes such as the cell cycle and NF-κB signalling, as well as nuclear transcriptional activities. These effects could significantly impact immune-inflammatory responses and the function of pigment cells in individuals with vitiligo. The abnormal activation of KEGG pathways such as the NF-κB signalling pathway and the Epstein–Barr virus infection pathway, as well as the activation state of immune cells, are also closely related to the pathogenesis of vitiligo. We verified the expression of the hub genes in blood via qRT–PCR. In addition, *PSMD13* significantly decreased in the lesioned skin of vitiligo patients, suggesting that *PSMD13* is involved in the skin lesion development process through the NOD-like-receptor signalling pathway, but *HMGA1* did not significantly decrease in the lesioned skin. Our study explored the potential of *HMGA1* and *PSMD13* as markers for vitiligo; these results validate our findings and have practical implications for the diagnosis and treatment of vitiligo. Future research should elucidate the specific roles of these molecules and pathways in the development of vitiligo to provide a stronger theoretical foundation for the development of new treatment strategies.

This study identified PSMD13 and HMGA1 as potential hub genes linked to vitiligo using integrated bioinformatics and experimental validation. While the exact immune mechanisms of PSMD13 are unclear, our findings lay a strong foundation for future studies on its functional role, especially in the Nod-like receptor signaling pathway. Furthermore, although the module-trait correlations in WGCNA were only marginally significant (*P* = 0.06 and *P* = 0.07), downstream analyses, such as functional enrichment and immune cell association, strongly supported the relevance of these modules. These limitations underscore the exploratory nature of this study and highlight the necessity for further validation using larger cohorts and functional assays. Despite these constraints, this study provides valuable insights into the molecular mechanisms underlying vitiligo and suggests novel targets for future diagnostic and therapeutic approaches.

## 5 Conclusions

In summary, we identified two vitiligo-associated genes, *HMGA1* and *PSMD13*, via WGCNA and machine learning approaches on datasets GSE80009 and GSE90880. We verified this trend by collecting blood samples from vitiligo patients and conducting qRT–PCR analyses of the identified hub genes. Additionally, we gathered microarray datasets (GSE53146 and GSE75819) from affected skin areas of these patients along with a control microarray dataset (GSE205155) from healthy skin for comprehensive expression and GSEA analyses of the hub genes. Our findings indicate that *PSMD13* plays a role in the skin lesion development process via the Nod-like receptor signalling pathway, whereas *HMGA1* does not seem to be directly involved in skin lesions according to our data. Despite their potential importance, research into the specific functions of *HMGA1* and *PSMD13* within the context of vitiligo remains scarce. Our work underscores the importance of *HMGA1* and *PSMD13* in the pathophysiological mechanisms underlying vitiligo, potentially opening new avenues for both diagnostic and treatment strategies for this condition.

## Data Availability

Publicly available datasets were analyzed in this study. This data can be found here: https://www.ncbi.nlm.nih.gov/geo/.
